# From bench to farm: leveraging *Drosophila* as a model insect for agricultural pest management

**DOI:** 10.1080/19336934.2026.2749209

**Published:** 2026-10-21

**Authors:** Yao D. Cai, Joanna C. Chiu

**Affiliations:** Department of Entomology and Nematology, College of Agricultural and Environmental Sciences, University of California Davis, Davis, CA, USA

**Keywords:** Pest management, insecticide resistance, invasive pest, Drosophila melanogaster, Drosophila suzukii

## Abstract

The vinegar fly, *Drosophila melanogaster*, has contributed tremendously to advancing our understanding of the fundamentals of biology, from the chromosomal theory of inheritance to the survival of multi-cellular organisms in spaceflight. The use of this insect as a model organism has provided insights into all branches of life sciences, including agricultural and environmental sciences. In the context of integrated pest management (IPM), which often relies on species-specific natural history and phenology of pest insects and biological control, *D. melanogaster* flies have continued to be a powerhouse. In this review, we seek to summarize the use of the *D. melanogaster* model in studying insect biology relevant to IPM, with a focus on investigating the mechanisms underlying insecticide resistance. We sample case studies that adopt forward genetic approaches to reveal molecular underpinnings of IPM-relevant phenotypes and reverse genetic approaches that elucidate genotype-phenotype relationships. In particular, we highlight the contribution of *D. melanogaster* in understanding the biology and in developing IPM for the spotted-wing drosophila, also known as *D. suzukii*, a pest *Drosophila* species that devastates fruit production worldwide. Finally, we summarize recently developed research tools and resources in *D. melanogaster* that have potential utility for agricultural pest management.

## Introduction

Agricultural productivity is essential for the food security of the expanding world population [1]. While crop yield has witnessed dramatic increases in the past six decades [2], agricultural pests and pathogens present a consistent challenge to the yield and quality of agricultural production [3]. Global yield loss for major crops, including wheat, rice and maize, ranges from 10.1% to 41.1% [3]. Due to global climate change, yield loss due to insect pests is expected to increase by 10% to 25% per degree Celsius of surface warming [4].

Chemical insecticides have been the central approach in agricultural pest control, safeguarding agricultural productivity and economies [5,6]. However, heavy reliance on chemical insecticides poses concerns about sustainability. There has been a cumulative elevation in the number of insect species that display resistance to one or more insecticides, which has led to reduced insecticide efficacy [7]. Increasing insecticide usage exacerbates the development of resistance in pests (reviewed in [8]) and elevates the cost to growers. Increasing insecticide applications can also be prohibitive due to maximum levels of pesticide residue that can legally remain on food. Moreover, research has revealed both acute and chronic negative impacts of pesticide exposure to humans [9–11], non-target species [12] and ecosystems [13,14]. Therefore, continuous efforts to understand resistance development and other aspects of insect biology to reduce reliance on chemical pesticides have become increasingly urgent.

*Drosophila melanogaster* offers many advantages as a model organism in biology [15–17]. Its taxon placement makes it appropriate for understanding insect biology and toxicology (reviewed in [6,18,19]). *D. melanogaster* has continued to yield mechanistic insights in integrated pest management (IPM), a framework that goes beyond chemical control. Since the beginning of the 21^st^ century, global and national organizations have endorsed IPM as the paradigm for crop protection [20]. IPM is an ecological strategy to manage agricultural pests, involving integration of cultural, biological, genetic and chemical control. The goal of IPM is to maintain pest populations below an economic threshold while minimizing the risks to the environment and human health. This review seeks to summarize the use of the *D. melanogaster* model in understanding insect biology relevant to IPM, with a focus on insecticide resistance. We sample case studies that adopt forward genetic approaches to reveal molecular underpinnings of phenotypes such as insecticide resistance, as well as reverse genetic approaches that elucidate genotype‑to‑phenotype relationships. Specifically, we highlight the recent contribution of *D. melanogaster* in understanding the biology and IPM of the spotted-wing drosophila, *D. suzukii*, a pest species that invaded the continental US in 2008 and quickly became a keystone pest of fruit production worldwide within a span of a few years [21]. We describe studies characterizing sensory, seasonal and circadian biology of *D. suzukii* and explain how they contribute to the success of *D. suzukii* as an invasive pest. Investigations into the sensory biology of *D. suzukii* have revealed mechanisms underlying host plant selection, which provided insights into bait and trap design [22,23]. Studies on seasonal biology have informed the potential of this pest to adapt to seasonal changes in environmental conditions and improved modelling of its range expansion [24]. Finally, studies on circadian biology of *D. suzukii* have promoted time-of-day awareness for effective IPM [25]. To end the review with a forward-looking lens, we summarize research tools and resources developed in *D. melanogaster* that have strong potential for use in agricultural pest management.

## *Drosophila melanogaster* as a model for insect biology relevant to pest management

For over a hundred years, *D. melanogaster* has been at the forefront of biological investigation. Research using *D. melanogaster* has been enormously fruitful in understanding the principles of genetic inheritance, developmental processes, regulation of metabolism and physiology and molecular bases of behaviour [15,17]. Perhaps not surprisingly, *D. melanogaster* has also been a critical model in revealing molecular mechanisms that confer resistance to insecticides.

Early research by James Crow and others established *D. melanogaster* as a model for resistance studies [26,27]. Many insecticide target genes were cloned and characterized in *D. melanogaster*, such as *resistance to dieldrin* (*rdl*) [28], *paralytic* (*para*) [29] and *Shaker* (*sh*) [30,31]. These target genes are highly conserved in insects, carrying broad implications for the biology of non-*melanogaster* insect species. Tracking a series of studies that characterize Dichlorodiphenyltrichloroethane (DDT) resistance best illustrates the utility of *D. melanogaster*. Crow first reported DDT resistance in *D. melanogaster* in the 1950s [26,27]. Despite early studies characterizing the genetic loci conferring DDT resistance [32,33], it was not until 2002 that DDT resistance was pinpointed to a single gene. The ffrench-Constant lab showed that the DDT resistance phenotype was associated with elevated transcription of a cytochrome P450 gene, *Cyp6g1* [34]. They found *Cyp6g1* expression levels in dozens of field-collected strains correlated with the resistance phenotype. Leveraging the versatile genetic toolkit in *D. melanogaster*, they developed transgenic flies overexpressing *Cyp6g1* and established a causal link of genotype to the phenotype of DDT resistance. In addition to genetic evidence, Joussen et al. [35] provided biochemical evidence that *D. melanogaster* CYP6G1 protein can indeed metabolize DDT. Tobacco cells expressing *D. melanogaster* CYP6G1 displayed an increased capacity to reduce DDT to the less toxic chemical DDD. Through this series of ground-breaking work, the molecular underpinnings of the DDT resistant phenotype were finally resolved after five decades.

Because of the evolutionary conservation of the cytochrome P450 (CYP) superfamily, the detoxification function of CYP6G1 discovered in *D. melanogaster* subsequently shed light on cytochrome-based resistance in other insects [36,37] (Figure 1). To reveal species-specific functions of CYPs, the ease of transgenesis in *D. melanogaster* has proven its efficiency and utility. Zimmer et al. expressed brown planthopper CYP6ER1 in *D. melanogaster* [40] and identified key amino acid polymorphisms that promote the binding of CYP6ER1 with imidacloprid. This study linked sequence changes of brown planthopper CYP6ER1 to resistance, in addition to quantitative changes at the mRNA level reported in [34]. To facilitate the investigation of species-specific cytochrome P450s, McLeman et al. generated transgenic flies expressing P450s of over a dozen pest and pollinator species [41]. These tools have been used to assess cross-resistance and the efficacy of synergists for pest management [42] as well as the toxicology of bee pollinators [43]. In addition to specific macromolecules (i.e. DNA, proteins) that insecticides covalently bind, insecticides can exert toxicity by increasing cellular oxidative stress (reviewed in [44]). Oxidative stress causes damage to DNA, proteins and alters the redox state of metabolite profiles [45]. Therefore, insecticide resistance can also be assessed through oxidative damage of DNA, protein peroxidation and alteration of the redox state of metabolite profiles [45]. The multilevel impact of oxidative stress on pesticide-induced toxicity can be readily revealed using *D. melanogaster* [46,47]. *D. melanogaster* also has a well‑characterized metabolome (Flymet.org) that can unveil changes in metabolite profiles upon insecticide exposure.

**Figure 1.**
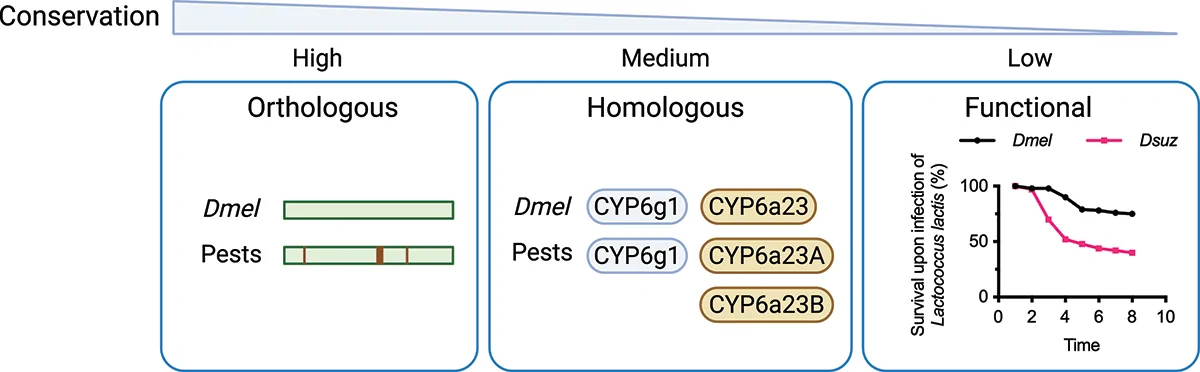
Levels of conservation between pests and *D. melanogaster*. Orthologous genes such as *resistance to dieldrin* (*rdl*) represent a high level of conservation, facilitating the translation of knowledge from *D. melanogaster* to pest species. Homologous genes such as members of the cytochrome P450 superfamily, represent a medium level of conservation, which benefit from validation of pest gene functions. Genes encoding CYP6g1, CYP6a23A and CYP6a23B are based on *D. suzukii* [38]. Response to bacterial infection represents a low level of conservation, which often requires parallel studies in pest species. Survival curves of *D. melanogaster* and *D. suzukii* upon *Lactococcus lactis* infection were adapted from Bing et al. [39].

When new modes of pesticide resistance emerge, *D. melanogaster* can be rapidly utilized for investigation. Sequestration resistance is a recently discovered mode of resistance, where overexpression of olfactory proteins binds and sequesters insecticides, in addition to causing behavioural avoidance of insecticides (reviewed in [48]). Conventionally, chemosensory proteins (CSPs) and odorant-binding proteins (OBPs) are olfactory proteins expressed in chemosensory structures to enable chemical communications [49]. Interestingly, overexpression of olfactory proteins has been observed in pyrethroid-resistant *Anopheles gambiae* mosquitoes [50,51]. Many studies have now leveraged genetic tools in *D. melanogaster* to establish a causal link between CSP levels and insecticide resistance in a number of pest species, such as cotton aphids (*Aphis gossypii*) [52,53] and diamondback moth (*Plutella xylostella*) [54].

*D. melanogaster* also promotes our understanding of biological control to a certain extent, despite the host specificity exhibited by individual pest species. Biological control reduces the population density of pest species by leveraging living organisms, such as predators, parasites, pathogens and symbionts [20]. For example, the *D. melanogaster*-parasitoid wasp system uncovered molecular mechanisms by which host biology is manipulated by the wasp to achieve reproductive success [55]. This could inform parasitic strategies that may be conserved in other host-parasitoid systems. Accumulating research has also examined host-pathogen interactions using *D. melanogaster*, in aspects including behavioural avoidance and immune responses (reviewed in Lu and Leger, 2016[56, 57]). Nonetheless, host specificity can limit the transfer of knowledge to non-*melanogaster* species. Even within the same genera, the pathogenesis of dozens of bacterial strains has been observed to exhibit differential survival in *D. melanogaster* and *D. suzukii* [39]. Limited efficacy of *D. melanogaster* parasitoid wasps was also found against *D. suzukii* [55]. Finally, studies on *D. melanogaster* behavioural ecology can provide insights into the behaviour of pest species towards improved pest management including biological control (e.g. [58].).

## Leveraging *Drosophila melanogaster* to elucidate phenotype-to‑genotype relationship in insecticide resistance

The phenotype‑to‑genotype approach, i.e. forward genetics, investigates the genetic basis of insecticide resistance identified in the field (Figure 2). Upon field collection and establishment of isofemale or isogenic lines in the laboratory, insecticide bioassays can be used to identify susceptible versus resistant lines. This approach was used in the DDT resistance research in the previous section. However, insecticide resistance is not always monogenic. With modern approaches such as high throughput sequencing that enables whole‑genome and/or transcriptome analysis, we have begun to appreciate the polygenic features underlying insecticide resistance. For example, transcriptomics analyses revealed overexpression of dozens of genes involved in metabolic and cuticular (penetration) resistance in *D. suzukii* resistant to spinosad and zeta-cypermethrin [59]. Using publicly available *D. melanogaster* knockdown mutants developed by the *Drosophila* community [60–62], this study functionally validated candidate *D. suzukii* resistance genes, demonstrating increased susceptibility when their expression was reduced. These findings highlight how *D. melanogaster* resources can expedite the establishment of causal links between phenotypes and genotypes in other pest species and inform resistance management strategies. The unveiled polygenic features of insecticide resistance offer valuable information for developing resistance management strategies. The study by Tabuloc et al. [59] also highlights that readily available knockdown *D. melanogaster* lines can expedite the establishment of a causal link of phenotype to genotype in other pest species.

**Figure 2.**
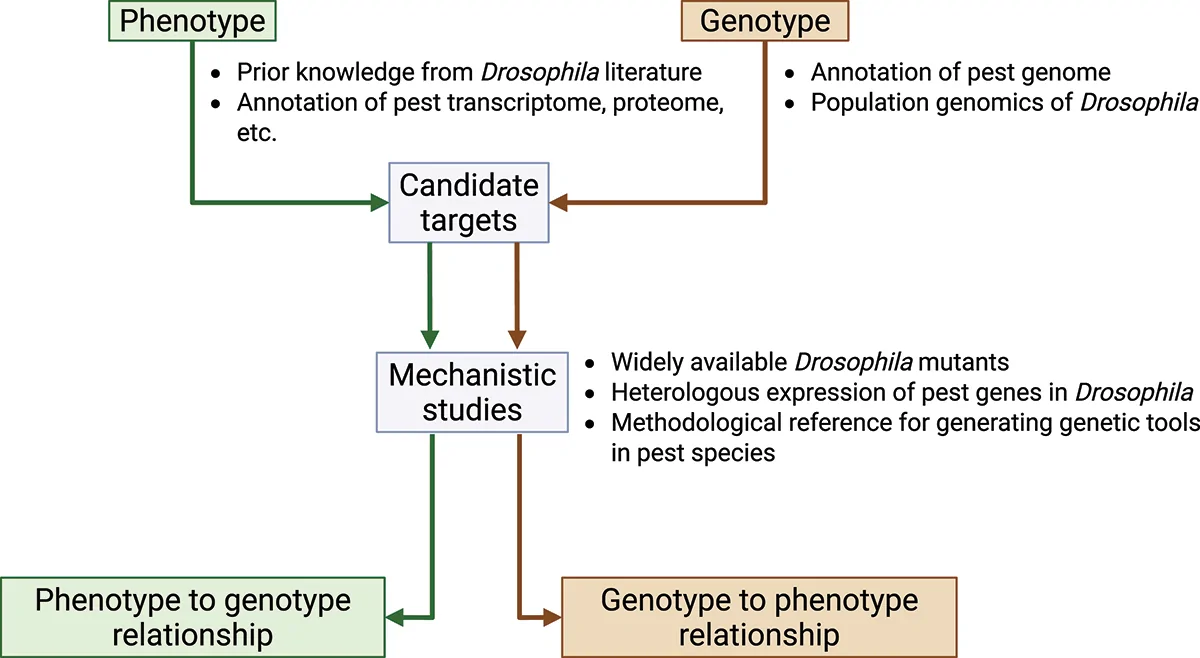
Workflow of using *D. melanogaster* to elucidate phenotype‑to‑genotype relationships as well as genotype‑to‑phenotype relationships in pests. Green arrows indicate the workflow of phenotype‑to‑genotype relationships; brown arrows indicate the workflow of genotype‑to‑phenotype relationships.

## Leveraging *Drosophila melanogaster* to reveal genotype-to‑phenotype relationship in insecticide resistance

The genotype‑to‑phenotype approach, i.e. reverse genetics, characterizes the phenotypic consequences when a gene is modified (Figure 2). The drastically reduced cost of DNA sequencing largely promotes the approach of probing the genotype prior to phenotype, making reverse genetics an effective way to understand mechanisms underlying insecticide resistance [63]. *D. melanogaster* was one of the first eukaryotic species to have its genome fully sequenced [64]. Remarkably, the *Drosophila melanogaster* Genetic Reference Panel (DGRP) [65] reported the genomes of 168 inbred *D. melanogaster* lines, presenting a rich resource for generating hypotheses of how genetic variants result in differences in phenotypic traits. For example, Remnant et al. identified gene duplication with point mutations of *Rdl* in DGRP lines [66], a gene that encodes a GABA-gated chloride channel subunit that is the target of multiple insecticides [67]. The permanent heterozygosity of *Rdl* alleles was shown to provide resistance to cyclodiene while reducing the temperature-sensitive fitness cost. This raised considerations for the continued use of insecticides targeting *Rdl* in several pest species, including pea aphids [68] and cotton bollworm [69]. One can also leverage DGRP for untargeted analyses, such as genome-wide association studies (GWAS). This has elucidated the quantitative and polygenic nature of resistance to insecticides including organophosphate insecticides [70], imidacloprid [71] and DDT [72]. Since transcriptome data are available for DGRP lines [73], GWAS can also be reinforced with transcriptome‑wide association study (TWAS), as reported by Denecke et al. [71]. Moreover, expanding numbers of species with genomes sequenced in the family Drosophilidae (12 genomes in 2007; 101 genomes in 2021) has facilitated the delineation of *melanogaster*-specific versus conserved mechanisms that can translate to the understanding of insect biology [74,75]. This will be further discussed in the next section.

## *D. melanogaster* elucidates the biology and management of *Drosophila suzukii*, a global fruit pest

Perhaps the best examples of harnessing *D. melanogaster* to study the biology of a pest species are from *D. suzukii*, a closely related species (Figure 3). *D. suzukii*, originally documented in Southeast Asia, has invaded over 50 countries and caused global crop damage [21]. Many *Drosophila* species, including *D. melanogaster*, prefer fermented fruits, which have no commercial value. In contrast, female *D. suzukii* possess serrated ovipositors and can lay eggs inside soft-skinned fruits, thereby damaging ripening and marketable fruits in the process [76]. The economic impact of *D. suzukii* is most severe in organic fields, mainly due to limited choices of insecticides [21]. The intensive use of chemical insecticides has been the primary approach for managing *D. suzukii*, prompting concern about resistance development over the past decade [77–79]. Indeed, several studies have now reported insecticide resistance in *D. suzukii*, significantly raising the concerns about a further decreased repertoire of insecticides [79–82]. Therefore, there is a pressing need to understand the biology of *D. suzukii* as a prerequisite for successful IPM of pest species. Since the invasion of *D. suzukii* into the continental US in 2008, *D. melanogaster* has facilitated and expedited our understanding of *D. suzukii* biology in many ways, which will ultimately aid in the development of better management strategies for this devastating pest.

**Figure 3.**
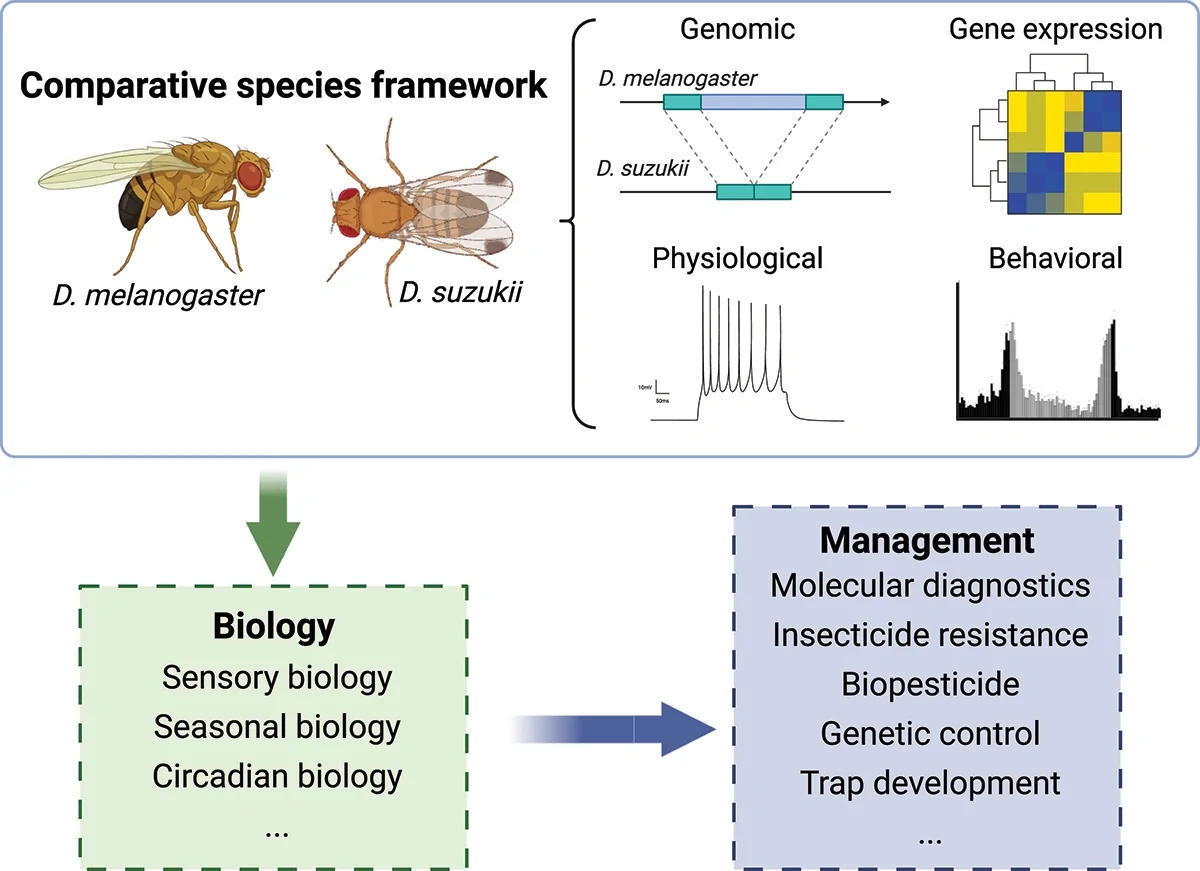
*D. melanogaster* elucidates the biology and management of *D. suzukii* through a comparative species framework including genomic, gene‑expression, physiological and behavioural analyses, to understand pest biology and management.

### Genomics

Genomic data have been used to understand the genetic underpinnings of pest species. *D. melanogaster*, together with other *Drosophila* genomes, served as references for gene annotation of the *D. suzukii* genome [83,84]. As compared with gene fragments [85], population analyses with whole‑genome data revealed invasion history and local adaptation that can inform the development of control methods (e.g. [86–88]). Genomic data can also facilitate the design of species diagnostics in the monitoring of field populations. Using a comparative genomics approach, molecular diagnostics were developed for the identification of *D. suzukii* at the larval stage, when morphology-based identification is difficult [89–91].

### Sensory biology

*Drosophila suzukii* evolved distinct chemo- and mechano-sensing mechanisms that shifted its niche towards ripening fruits [22,23]. As fruits ripen and overripen, physical and chemical properties continue to change, including softness, sugar content, bitterness, acidity, CO_2_ production, odour and alcohol [92]. A comparative species framework leveraging behavioural, electrophysiological, gene expression, genomics and molecular genetic approaches has proved fruitful in understanding the preference of D. suzukii for egg laying on ripening fruits over fermented ones[22,93–95]. Early works on the molecular mechanisms of odorant receptors in *D. melanogaster* revealed the function of the odorant receptors gene family in distinct odour sensory neurons [96,97]. Compared with *D. melanogaster*, *D. suzukii* flies possess 20% fewer bitter-sensing sensilla, the major taste organ of the head [22], indicating reduced deterrence of ripening fruits that contain abundant bitter compounds. Indeed, bitter-sensing *D. melanogaster* genetic mutants shift the egg‑laying preference toward ripe fruit. For sugar response, expression of several sugar receptor genes is reduced in the *D. suzukii* sensilla compared with *D. melanogaster* [23]. This correlates with the lower sugar response reported in *D. suzukii* sensilla. Overall, these chemosensory changes contribute to the weaker preference of *D. suzukii* for sweeter and overripe fruits. Furthermore, sugar perception is enhanced in *D. suzukii*, which suggests that the low level of sugar in ripe fruits is more likely to trigger oviposition in *D. suzukii* than in *D. melanogaster* [94]. During the onset of ripening, fruits exhibit ramping respiration rates and produce CO_2_, which is not favored by many *Drosophila* species (e.g. [93]). *D. suzukii* flies exhibit greater CO_2_ tolerance compared with *D. melanogaster* [95]. Expressing *the D. suzukii* CO_2_ receptor in *D. melanogaster* shifts its egg‑laying preference to ripe fruits, establishing a causal link of CO_2_ tolerance and egg‑laying preference. A more recent study revealed that the gained chemosensitivity to fruit-ripening esters in *D. suzukii* is due to a collective shift of SNPs, copy number changes and co‑expression of a few odorant receptors [98]. In addition to chemosensation, certain mechanosensory channel genes are elevated in the sensilla of *D. suzukii*, resulting in a higher preference for stiff (ripe fruits) over soft substrates (overripe fruits) [99].

The understanding of *D. suzukii* sensory biology, together with chemical compound screening, has the potential to improve targeted trap design for *D. suzukii* without capturing non-target *Drosophila* species [100]. Commercially available lures can capture over 40% non-target species, requiring time and labor for sorting *D. suzukii* for population‑monitoring purposes [101]. Combining comparative species analysis with screening of chemicals from fruits (e.g. [102]) is therefore expected to achieve more effective species-specific trap design. Since *Drosophila* species exhibit species-specific mutualistic relationships with yeasts, assessment of attraction to volatile organic compounds from yeasts also provides candidates for trap design [58,100]. Chemical screening approaches may also guide the design of volatile food-grade gum [77,103] as an environmentally friendly attract-and-kill strategy for *D. suzukii* management.

### Seasonal and circadian biology

The ability to tolerate cold, harsher winter contributes to the success of *D. suzukii* in a wide range of climatic conditions. Investigating overwintering capability was defined as one of the early top priorities for *D. suzukii* biology [24]. *D. melanogaster* has again demonstrated its value in this research area. Reproductive dormancy was first documented in *D. melanogaster* [104]. Using quantitative trait mapping in combination with transgenic approaches, Schmidt et al. [105] identified *couch potato* (*cpo*), the first gene responsible for reproductive dormancy. *cpo* expression level is associated with winter acclimation in *D. suzukii* [106]. Population genomics also suggested a strong association of SNPs in *cpo* with invasion success in non-native climatic conditions of *D. suzukii* [107]. Future research may leverage *D. melanogaster* genetics to reveal the potentially conserved mechanisms by which *cpo* regulates reproductive dormancy.

We and others investigated environmental cues that modulate the seasonal biology of *D. suzukii* [108,109]. In response to short photoperiod and cold temperature, *D. suzukii* increases cold tolerance and enters reproductive dormancy. Leveraging *D. melanogaster*, we identified the roles of circadian clocks in regulating reproductive dormancy. We showed that the circadian clock gene *timeless* (*tim*) senses photoperiodic changes through the light sensitivity of the TIM protein [110]. This, in turn, stabilizes EYES ABSENT (EYA) proteins, which promotes the incidence of reproductive dormancy through endocrine systems. Our follow-up studies further revealed that the cold-induced TIM-short and cold (TIM-SC) isoform, produced through alternative splicing, increases EYA level [111,112]. These studies linked the circadian clock to seasonal reproductive dormancy. Other than *tim*, we found that temperature-sensitive alternative splicing of *Clock* (*Clk*) is also required for a proper temperature response [113]. Inspection of genomic *tim* and *Clk* of several *Drosophila* species indicates that other *Drosophila* species, including *D. suzukii*, also have potential splicing mechanisms that regulate reproductive dormancy. In fact, *the tim-SC* transcript has been shown to exist in a cold-adapted fly, *D. Montana*, supporting this speculation [114].

Similar to many other insects, *D. suzukii* displays circadian rhythms in physiology and behaviour. Steed et al. [115] introduced the concept of ‘chronoculture’ to harness the daily timing of agronomic approaches and plant circadian rhythms for improving crop yield and sustainability. Understanding the circadian biology of pests is one key aspect of chronoculture. Just like *D. melanogaster*, *D. suzukii* exhibits the highest locomotor activity around dawn and dusk [116]. This correlates with the highest number of individuals captured in monitoring traps [117] and the highest infestation rate in fruits [118]. Taking chemical control as an example, if detoxification of *D. suzukii* also exhibits daily rhythms, the overlapping time window would be optimal for insecticide efficacy. Indeed, the mRNA expression levels of multiple detoxification genes exhibit daily rhythms and may be associated with the highest toxicity of *D. suzukii* at dawn in response to exposure to malathion [25]. Therefore, insecticide spray applications may be best implemented during early morning, especially for chemicals that are less stable. We expect the versatile genetic tools and well-established molecular assays in *D. melanogaster* will continue to facilitate investigations into *D. suzukii* circadian biology.

### Genetic toolkit

Several endeavours have expanded the genetic toolkit in *D. suzukii* inspired by that of *D. melanogaster*. The Handler lab and the Prud’homme lab first adapted the *PiggyBac*-based transgenesis from *D. melanogaster* [60] to *D. suzukii* [119,120]. This has contributed to the understanding of the shifted niche of *D. suzukii* towards ripening fruits [94,120]. To avoid potential position effects and mutations, the Handler lab also adapted recombinase-mediated cassette exchange (RMCE) from *D. melanogaster* [121–123]. The Scott lab introduced heritable mutations in *D. suzukii* to allow phenotypically trackable genetic markers [124,125]. The expanded genetic toolkit in *D. suzukii* not only facilitates biological research but also pushes forward the application of genetic control (reviewed in [126]). It is worth noting that most genetic strategies in *D. suzukii* also benefited from the pioneering work in *D. melanogaster* (e.g. [127,128]). Finally, research employing *D. melanogaster* continues to expand the horizon of genetic control in pest species. For example, an allelic drive system can replace a resistant allele with a wild-type susceptible one, thereby specifically reversing insecticide-resistant state to the native susceptible populations [129].

## *Drosophila melanogaster* as a powerhouse for development of novel insecticides

Harnessing *D. melanogaster* resources can also drive the development of novel insecticides through high‑throughput screening, especially for dipteran pests. Various behavioural assays have potential for efficacy screening, including lifespan, climbing ability, circadian rhythms, growth and reproduction (reviewed in [130]). Forward genetic approach can then be employed to reveal underlying mechanisms. The same behavioural paradigms can also evaluate sublethal effects of insecticide exposure. These low‑dosage, chronic or multi-generational impacts have posed ecological [13,131–133] and public health concerns [134,135].

Recently developed adult *Drosophila* cell lines [136,137] may further expedite novel insecticide development. Of these cell lines, we expect neuronal and intestinal cell lines to be particularly useful for screening and mechanistic studies, because the majority of insecticides target nervous and digestive systems. For example, an intestinal cell line can enable the study of insecticide detoxification, such as plant-derived compounds [138] or bacterial toxins [139]. Cell line models can reveal cell-type‑specific resistance mechanisms to complement whole‑insect models, such as the expulsion of xenobiotics by ATP-binding cassette (ABC) transporters in the gut [140].

## Conclusion and perspectives

*D. melanogaster* research has made enormous contributions to agricultural pest management. Tools and resources have been growing rapidly in the *Drosophila* research community, such as *the Drosophila* computational brain model [141] and single-nucleus transcriptomic atlases of embryos [142], larvae [23] and adults [143,144] that offers single-cell resolution. The larval [145] and adult connectomes [146,147] may further reveal the mechanisms conferring insecticide resistance, as evident in a study uncovering the spatial organization of acetylcholine receptors nAchRs, major targets of widely used insecticides [148]. Combined with deep learning-based prediction tools such as AlphaGenome [149] for transcriptional regulation and AlphaFold [150] for protein structures of variants encoded by resistant alleles, we anticipate that the *D. melanogaster* system will continue to provide significant insights into agricultural pest management.
